# The Comparative Study on Molecular Phylogenetics and Development of Microsatellite Primers From Durian Chloroplast Genomes

**DOI:** 10.1002/ece3.72245

**Published:** 2025-10-07

**Authors:** Tran Gia Huy, Pham Thanh Phuc, Nguyen Pham Anh Thi, Tran Van Be Nam, Do Tan Khang

**Affiliations:** ^1^ Institute of Food and Biotechnology Can Tho University Can Tho Vietnam

**Keywords:** chloroplast genome, *Durio zibethinus*, microsatellite DNA, phylogenetic

## Abstract

Durian, or *Durio zibethinus* , is an exotic fruit with high economic value in Southeast Asia. This study aims to determine the genetic variants based on the chloroplast genome between species and cultivars in the *Durio* genus and design the molecular marker based on microsatellite resources. The research was conducted by collecting the data of 8 chloroplast genomes followed by the genome structure and organization analysis, variants analysis, phylogenetic relationship analysis, and possibly, primer designing. The results illustrate that regions with high Pi values for the nucleotide diversity analysis were the regions in *ycf*1, *psb*Z‐*rps*14, *acc*D, and *rpl*33‐*rps*18. Although the nucleotide diversity analysis had shown various hotspots from the current data of complete chloroplast genomes of the *Durio* genus, the *acc*D gene was shown to have a high Pi value between species but failed to distinguish between variants of the same species. Furthermore, 13 primer SSR regions were selected from 135 SSRs, and the corresponding primer pairs were designed for amplification. *Durio zibethinus* cv. Ri6 has a close relationship to other *Durio zibethinus* cultivars but has high genetic divergence from other *Durio* genus species. Both the phylogenetic trees constructed from SNPs/InDels and from CDS data produced concordant clustering results, revealing consistent genetic relationships among species within the genus Durio. The cp genomes are stated to be a useful tool in studying the phylogenetic relationship. In conclusion, the cp genomes are a useful tool and could be further utilized for molecular markers designing and the evolution history of the *Durio* genus.

## Graphical abstract

Based on the chloroplast genomes, the phylogenetic relationship of durian cultivars was visualized. Furthermore, 13 SSR primer pairs were designed and could be applied for genetic diversity and genotyping studies.

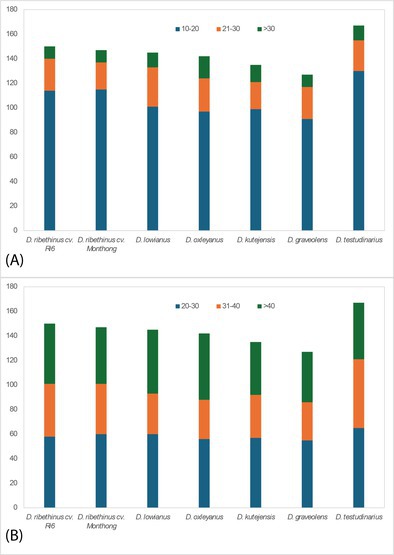

## 1 Introduction

The chloroplast genome has emerged as a powerful tool in the study of plant evolution, phylogeny, and genetic diversity. Chloroplasts, the organelles responsible for photosynthesis in plant cells, possess their genomes, which are typically circular and encode a set of essential genes involved in photosynthesis, transcription, and other vital cellular processes (Jensen and Leister 2014). Due to the relatively conserved nature of chloroplast genomes across plant species, they have become valuable in phylogenetic studies, enabling researchers to trace evolutionary relationships and species divergence with high precision (Caycho et al. 2023; Machado et al. 2022; Xue et al. 2019). One of the significant advances in plant molecular biology is the use of the chloroplast genome as a newly transferred plasmid as many occurrences have been observed to have transferred the gene from the chloroplast genome into the nuclear genome, but this phenomenon has not been researched deeper into its application or mechanism (Filip and Skuza 2021; Stegemann et al. 2003). By integrating the entire chloroplast genome or specific regions of it into phylogenetic models, researchers can generate more accurate and resolved phylogenies.

Chloroplast genomes have also gained prominence as sources of species‐specific markers and biomarkers or barcoding for elite mother plants as the chloroplast genomes are maternal inheritance, the genetic material inherited from the mother plant (She et al. 2022; Teske et al. 2020). Species‐specific markers derived from the chloroplast genome are highly valuable in distinguishing between closely related species and in identifying hybrids. One of the key applications of chloroplast genome analysis is in studying the genetic differences between cultivated and wild plant species. By looking into the genetic profiles of domesticated plants and wild‐type plants, we can observe how the domestication process affects and changes the inheritance material between these plants in the same species or genus (Daniell et al. 2016; Moner et al. 2020). These differences are evident in both the chloroplast genome and nuclear genome, reflecting the impact of domestication and selective breeding on the genetic makeup of cultivated plants. Chloroplast genome analysis provides a detailed understanding of these genetic differences, offering insights into the evolutionary history of durian and the genetic basis of traits important for cultivation. This information is critical for developing strategies to conserve the genetic diversity of wild durian species, which may harbor valuable traits that could be reintroduced into cultivated varieties to improve resilience and adaptability.

Durian, often referred to as the “king of fruits,” is an exotic tropical fruit that holds immense economic and cultural significance in Southeast Asia (Ketsa et al. 2019). In Vietnam, the durian ( *Durio zibethinus* L. (1774)) is highly economic, and the varieties that the farmer mainly cultivates and commercializes are Sua Hat Lep, Monthong, Ri6 (Hau and Hieu 2017). Not only is durian popular in Southeast Asia but also popular in the international marketplace (Ketsa et al. 2019). According to the Ministry of Agriculture and Rural Development of Vietnam (2023), the area for cultivating durian of Vietnam was over 150,000 ha, with a yield of approximately 1.2 million ton, and 50% of the yield was exported to the international marketplace. The most widely cultivated species, *Durio zibethinus* , is prized for its unique flavor, aroma, and nutritional value. The durian industry contributes significantly to the economies of countries like Thailand, Malaysia, and Indonesia, where the fruit is a major export commodity (Ketsa et al. 2019). In recent years, the global demand for durian has surged, driven by increasing popularity in China and other international markets. Beyond its economic value, durian has considerable scientific value, particularly in the fields of plant genetics, breeding, and conservation biology. The genus *Durio* comprises approximately 30 species and over a hundred cultivars, many of which are endemic to the rainforests of Southeast Asia (Ketsa et al. 2019). Although *Durio zibethinus* is the only species widely cultivated for its fruit, other species such as *Durio graveolens*, *Durio oxleyanus*, and *Durio kutejensis* have also garnered interest for their unique fruit characteristics and potential for hybridization (Thorogood et al. 2022). The genetic diversity within the genus *Durio* makes it a fascinating subject for scientific research, particularly in understanding the evolutionary processes that have shaped the diversity of fruit traits, such as aroma, flavor, and seed size, across different species. Not only that the difference between wild durian species with its numerous under‐species cultivars raises a highly biodiversity within just the *Durio* genus. In addition, cultivated durians often exhibit reduced genetic diversity compared to their wild counterparts, a consequence of selective breeding for specific traits such as fruit size, flavor, and yield. In the case of durian, with the propagation methods of grafting the branch of durian cultivars ( *D. zibethinus* ) capable of producing high quality durian fruit with wild durians (*D. oxeylanus* or *D. graveolens* ). This technique not only produce high yield durian plants with significant genetic divergence but also cuts the down needed to wait for the durian seedlings to sprout (Hasanah and Mariana 2024). *D. zibethinus* cultivar Ri6, is an exclusive durian cultivar in Vietnam which is made by using the grafting technique by a Vietnamese farmer. According to Huy et al. (2024), although being in the same species, the cultivar Ri6 has some noticeable differences that make it separate to the Monthong cultivar, but not so different that it is still related close enough to be on the same branch of the phylogenetic tree.

Although the durian cultivar Ri6 or *Durio zibethinus* cv. Ri6 is considered the best durian in Vietnam with high economic value, but not much research has been conducted to study the genetic information. Although the physiological appearances are noticeable between different varieties of durian, these characteristics are easily influenced by the growing environment and therefore, are not stable in determining. But when it comes to DNA, it is much more stable as it is the genetic information located inside the cells and cannot be influenced by environmental conditions.

One of the primary areas of scientific interest in durian research is the study of its chloroplast genome, which provides insights into the evolutionary history and phylogenetic relationships among durian species. Chloroplast genome sequencing has revealed significant variation in the genome structure and gene content among different durian species. These variations are not only important for resolving phylogenetic relationships but also for understanding the genetic basis of traits that are unique to certain species. For instance, the distinct aroma of durian, which is a key factor in its commercial value, has been linked to specific genes involved in sulfur metabolism. Comparative analysis of chloroplast genomes across durian species can help identify the evolutionary origins of these traits and their role in species differentiation (Kanzaki et al. 1998). Based on the current data of Durian chloroplast genomes from NCBI database, this study aims to identify the variants for both gene coding and repeat regions; visualize the phylogenetic relationship of *D. zibethinus* cv. Ri6, an indigenous Vietnam cultivar, among other species of the *Durio* genus as well as other species of Malvaceae family.

## 2 Materials and Methods

### 2.1 Data Collection

The data of *Durio zibethinus* cv. Ri6 chloroplast genome was utilized from our last published research, which was extracted, sequenced and sent into GenBank database. No genome extraction or sequencing was performed in this research.

For the collection of other cp genomes, the data was collected by the latest dates this research has been conducted. The data was collected on NCBI (National Center for Biotechnology Information (https://www.ncbi.nlm.nih.gov)) based on the phylogenetic relationship from Plants of the World Online website (https://powo.science.kew.org) of Royal Botanic Gardens Kew.

The criteria for the data collection was: (1) The collected genomes are references sequences with the “NC” tag in the accession number for all cp genomes of Malvaceae; (2) For the genus in Malvaceae that have the number of reference sequences equal or over three, three sequences of different species was collected, for the genus that have less than three reference sequences, all were collected. (3) All complete cp sequences of the species of *Durio* genus were collected whether or not they had the “NC” tag or not. The genomes are cp genomes of the Malvaceae family and two species from Dipterocarpaceae family were used as an outgroup when reconstructing the phylogenetics tree; (4) The cp genomes are completed genome sequences.

All nine (up‐to‐date) completed chloroplast genomes of *Durio* species were collected for the analysis, including *Durio ribethinus* cv. Ri6 (OR731187.1), *Durio ribethinus* cv. Monthong (NC_036829.1), *Durio ribethinus* cv. Monthong (MT321069), *Durio testudinarius* (PP668204.1), *Durio oxleyanus* (NC_064728), *Durio lowianus* (PP668207.1), *Durio kutejensis* (PP668203.1), *Durio graveolens* (PP668202.1), *Durio dulcis* (NC_073110.1). For other species in the Malvaceae family, two to three available completed sequences of each group were collected. For the outgroup species, two species were selected from Dipterocarpaceae family, *Dipterocarpus turbinatus* (NC_041191.1), *Vatica odorata* (NC_054172), and *Neobalanocarpus heimii* (NC_046842.1).

### 2.2 Genome Structure and Organization Analysis

After collecting all the data, The gene content of such chloroplast genomes was annotated with the Geseq tool (Tillich et al. 2017). Then, the cp genomes were analyzed the structure for the size of each region of the cp genome, including Large‐single copy (LSC), Small‐single copy (SSC) and two Inverted Repeats (IRs). After that, the numbers of protein‐coding genes (PCGs), tRNA genes and rRNA genes were recorded. The GC content was also be collected as well as analyze the genes they are having in similarity and difference. The numbers of gene having one intron, or two introns were also collected.

### 2.3 Variants Analysis

#### 2.3.1 Nucleotide Diversity in Chloroplast Genomes of *Durio* Genus

To calculate the nucleotide diversity (Pi values) among the chloroplast genomes of *Durio* genus, DnaSP v6 had been used (Rozas et al. 2017). Before the software could be utilized, the alignment of nine species/cultivar were employed using MAFFT v1.1.2 with default parameters. Then the alignment file was exported as. meg or fasta file. If the aligned sequences have any ambiguous codes, the fasta file first needs to be converted by the tools on the DnaSP v6 to remove the ambiguous codes for the calculation. The aligned sequences were imported to the DnaSP for calculating the Pi values and performing sliding window analysis with the window size of 2000 and step size of 100 (Khoa 2022).

#### 2.3.2 SNPs/Indels Analysis

To detect the SNPs or indel variants, the coding sequences of all sampled species were first aligned using MAFFT v1.1.2 (Katoh and Standley 2013) with default parameters. Then, the tools find variation of SNPs/indels in Geneious Prime were implied with default options to search for the SNPs and indels of the alignment of selected. Then by selecting all the SNPs/indels andit extracting for further analysis. The information of types of polymorphisms (SNPs or indels), the location of those polymorphisms (IGS or CDS) were collected.

#### 2.3.3 Repeat Sequences Analysis

To analyze the repeat sequences, an Inverted Repeat (IR) in the cp genome was removed as the genetic sequences of two Inverted Repeat are identical but inverted so the number of inverted sequences were doubled if the two IRs are present.

#### 2.3.4 SSR Identification and Primer Design

To find short repeat sequences (SSRs), MISA (MIcroSAtellite identification tool) (Beier et al. 2017) webserver was utilized to find mono‐, di‐, tri‐, tetra‐, penta‐, and hexa‐nucleotide with the minimum length of 10, 6, 5, 5, 5, and 5 bp, respectively (default parameter). After collecting the SSR data, Primer3Plus tool (https://www.primer3plus.com/primer3plusPackage.html) was employed to design the primer for each SSR (Untergasser et al. 2012), with the amplicon size varied from 200 to 1000 bp. Then the NetPrimer tool of PREMIER Biosoft (http://www.premierbiosoft.com/NetPrimer/AnalyzePrimer.jsp) was employed to check the quality as well as the melting temperature (T_m_) of the primer, the criteria for choosing the primer pair was both of the primers need to have the rating score of at least 85. For those primer pair with one of the pair having the rating score above 95, the other pair can be choosing the rating score of at least 80. The designed primers were validated by amplification and visualization under 2% agarose electrophoresis. The target DNA fragments were amplified in a 30 μL PCR reaction. Each reaction mixture contained 15 μL of 2× master mix (Vazyme, China), consisting of *Taq* DNA polymerase, dNTPs, and buffer, 0.4 μM of both forward and reverse primers, 50 ng of template DNA, and nuclease‐free water to the final volume. The primer sequences are presented in Table 2. Amplification was carried out using a Mastercycler x50s thermal cycler (Eppendorf, Germany). The PCR thermal profile consisted of an initial denaturation at 95°C for 3 min, followed by 35 cycles of denaturation at 95°C for 30 s, annealing for 30 s, and extension at 72°C for 1 min, with a final extension at 72°C for 5 min. The gradient PCR was conducted to screen the optimal annealing temperature for each primer pair. The resulting PCR products were resolved on 2% agarose gels by electrophoresis at 50 V for 45 min, and the DNA bands were visualized using the GelDoc‐XR system (Bio‐Rad, USA). The amplicon size was verified using a 100 bp DNA ladder (Biohelix, Taiwan).

#### 2.3.5 Tandem Repeat Identification and Analysis

The Phobos program (Leese et al. 2008) in Geneious Prime was ultilized to the find long tandem repeats with the configuration of choosing perfect search model, enabling remove hidden repeat, minimum and maximum repeat length of 10 and 200, respectively.

### 2.4 Phylogenetic Relationship Analysis

To analyze the phylogenetic relationships of *D. zibethinus* cv. Ri6 with other species from *Malvaceae* family, nine completed cp genome of cultivars and species of Malvaceae family were collected and three completed cp genome of two species from Dipterocarpaceae family, *Dipterocarpus turbinatus* (NC_046842.1), *Vatica odorata* (NC_054172), and *Neobalanocarpus heimii* (NC_041191.1). Both of the selected family is in the Malvales order were collected and used for reconstructing the phylogenetics tree.

Two phylogenetic trees were be reconstructed based on different objective: the first phylogenetic tree was reconstructed based on PCGs while the second phylogenetic tree was reconstructed based on the SNPs regions. To reconstruct two different phylogenetic trees, two types of data are needed. For the PCGs phylogenetic tree, the coding sequences of all sampled species were extracted and aligned using MAFFT v1.1.2 (Katoh and Standley 2013) with default parameters. The intergenic spacers were removed using Geneious Prime, and then the sequences were concatenated to produce a complete PCGs database. For the SNPs phylogenetic tree, the coding sequences of all sampled species were extracted and aligned using MAFFT v1.1.2 (Katoh and Standley 2013) with default parameters. Then, the SNPs from both the intergenic spacer and CDS were extracted as well as removing non‐SNP sequences for better processing. Before reconstructing the Maximum Likelihood phylogenetic tree, the optimal evolutionary model for two phylogenetic trees was calculated using jModelTest v2.1.10 (Santorum et al. 2014). Then, the IQ‐TREE webserver was employed to reconstruct the phylogenetic trees by the maximum likelihood method with 1000 replication bootstraps (Minh et al. 2020). FigTree v1.4.4 was utilized to visualize the resulting tree (Drummond et al. 2012).

## 3 Results and Discussion

### 3.1 Genome Structure and Organization Analysis

According to the NCBI database, only eight over 24 species of *Durio* genus have been sequenced and published the complete chloroplast genome on to the GenBank database of NCBI, of which two cultivars of the *Durio zibethinus* have been uploaded on the GenBank. The cp genome sizes of the species of *Durio* genus are varied between 163,974–166,346 bp. The average GC content of eight sequenced cp genome of *Durio* species is 35.8% with the highest GC content is 35.9% of *Durio oxleyanus* (NC_064728) and *Durio graveolens* (PP668202) and the lowest GC content is 35.6% of *Durio testudinarius* (PP668204). The average GC content of LSC, SSC and IRs are 33.7%, 30.8% and 42.4%, respectively. All species in the *Durio* genus have the same amounts of protein‐coding genes (PCGs), tRNA genes and rRNA genes, which are 83, 37 and eight, respectively. Within that, 15 genes have one intron (*ndh*A, *ndh*B, *atp*F, *pet*B, *pet*D, *rpl*2, *rpl*16, *rpo*C1, *rps*16, *trn*A‐UGC, *trn*G‐UGC, *trn*I‐GAU, *trn*K‐UUU, *trn*L‐UAA, and *trn*V‐UAC) and 2 genes have two introns (*clp*P, and *ycf*3). Within the IRs, five PCGs (*ndh*B, *rps*7, *rps*12 and *ycf*2), four rRNA genes (*rrn*5, *rrn*4.5, *rrn*23, *rrn*16), and seven tRNA genes (*trn*N‐GUU, *trn*R‐ACG, *trn*A‐UGC, *trn*I‐GAU, *trn*V‐GAC, *trn*L‐CAA and *trn*I‐CAU) were duplicated.

Although being in the same species as *Durio zibethinus* , the cultivar Ri6 has a larger size of cp genome when compared to the cultivar Monthong. The major changes that lead to the differences in size are the inverted repeats. The cultivar Ri6 has 24,185 bp per repeat, which when compared in pairs leads to 918 more bp than cultivar Monthong. Other small changes are a slight increase of about 400 bp in the LSC region and the addition of one extra bp in the SSC region. Not only that, but the cultivar Ri6 is also larger than other cultivars of the same species. The IRs of the Ri6 are the largest IRs among other species in the *Durio* genus, with noticeable differences in size of bp, which leads to the potential region for searching for biomarkers and consequently, designing primers for the identification of *D. zibethinus* cultivar Ri6.

Although not mentioned in Table 1, there are two other species in the *Durio* genus that have lost one IR phenomenon, which are *Durio dulcis* (NC_073110.1) and one cultivar of *Durio zibethinus* cv. Monthong (MT321069). This phenomenon has led to the reduction of about 20 kbp in the size of the cp genome of these two cases. Consequently, the loss of one IR copy leads to the loss of PCGs, tRNA genes, and rRNA genes in this region. Although this phenomenon is not new in the history of the plant cp genome, it is uncommon in the *Durio* genus or in the Malvaceae family overall. But since not all cp genomes of the species in the *Durio* genus have been sequenced and completed, the uncommon nature of this phenomenon might be resulting from this. The cases might change when all the cp genomes in this genus have been sequenced.

**TABLE 1:** *Durio* species and their accession number, length, and GC% of whole cp genome, LSC, SSC, and IR (bp).

Scientific name	Accession number	Length (bp)				GC%			
		CP	LSC	SSC	IR	CP	LSC	SSC	IRs
*Durio ribethinus* cv. Ri6	OR731187	165,304	96,115	20,819	24,185	35.7	33.6	30.7	42.1
*Durio ribethinus* cv. Monthong	NC_036829	163,974	95,704	20,818	23,726	35.8	33.6	30.8	42.5
*Durio lowianus*	PP668207	165,468	96,671	21,281	23,758	35.8	33.7	30.7	42.5
*Durio oxleyanus*	NC_064728	166,346	97,513	21,319	23,757	35.9	33.9	30.9	42.5
*D. kutejensis*	PP668203	165,149	96,533	21,010	23,803	35.7	33.6	30.7	42.3
*D. graveolens*	PP668202	164,233	95,559	21,312	23,681	35.9	33.8	30.7	42.6
*D. testudinarius*	PP668204	165,462	97,013	21,009	23,720	35.6	33.4	30.8	42.3

### 3.2 Variants Analysis

#### 3.2.1 Nucleotide Diversity in the Chloroplast Genomes of *Durio* Genus

**FIGURE 1:**
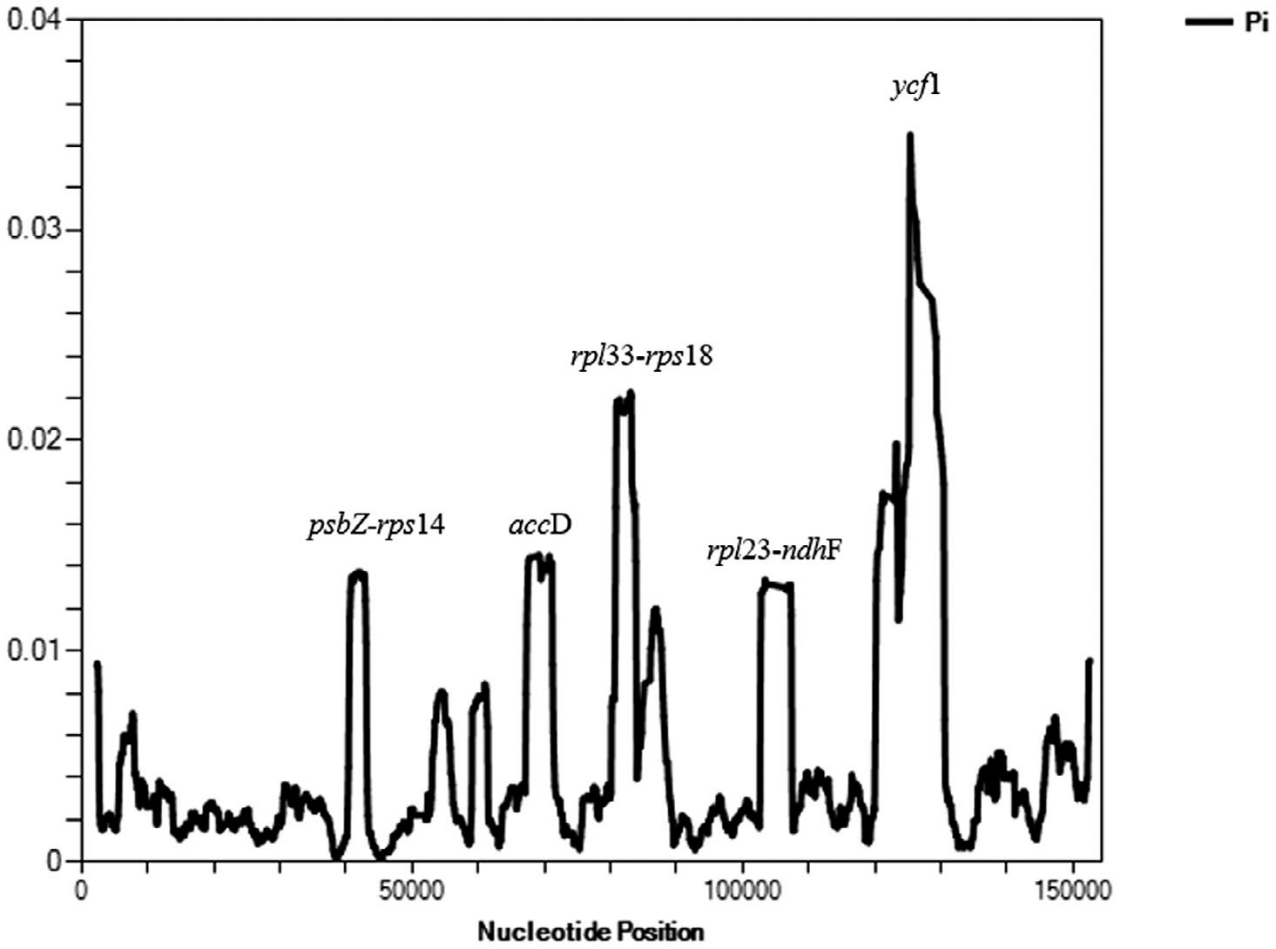
Sliding window analysis of the whole chloroplast genomes of *Durio* species. (window length: 2000 bp, step size: 100 bp) *X*‐axis, nucleotide position, *Y*‐axis: Pi value of each window.

From Figure 1, the nucleotide diversity analysis of current sequenced genomes has shown some statistical information of Pi value from the alignment analysis of their chloroplast genome. The highest Pi value recorded in sliding window analysis of *Durio* genus is > 0.04 at the junction region of SSC and IRa, or the region of the *ycf*1 gene. Regions with high Pi value noticeable from the figure include the region in *psb*C*‐rps*14, *rbc*L*‐acc*D, *rpl*33*‐ycf*3, *and ndh*F. These regions with high Pi value mean that there are noticeable differences among the durian species used in this analysis. Therefore, these peaks of high Pi value can be potential candidates for designing specific primers to distinguish different species in the genus or even different subspecies, cultivars in the species. In contrast, regions with low Pi value mean there are low to no differences between species within the same genus. In extent, these low Pi value regions can be used as a potential region of recognition for *Durio* genus in general.

#### 3.2.2 SNPs/Indels Analysis

After the alignment of seven *Durio* species, the tool to find the single nucleotide polymorphisms (SNPs) and insertions and deletions (indels) was utilized. As a result, a total of 5561 nucleotide locations with polymorphic properties of seven *Durio* species have been recorded. Within those 5561 polymorphic nucleotides, over 90% of the cases were indels (5122 nucleotides); the small percentage left belonged to SNPs with 449 polymorphic nucleotides. A large number of SNP loci are located on the *ycf*1 genes among seven *Durio* species, which have a total of 102 bp differences in SNP loci. Some small differences in the SSC regions among the *Durio* species are located in the *ndh*F and *ndh*D genes. For IRs, only the *ycf*2 gene was detected with 7 SNP loci. For LSC, although having many more genes with SNPs detected, most of them had only one SNP detected; genes like *acc*D and *rpo*B are genes in the LSC region that have more than one SNP locus, which are 26 and 5 bp, respectively.

Within the indel polymorphism cases, there is a big difference in the distribution between the amount of those indels in the Coding sequence (CDS) and intergenic spacer (IGS). For instance, the number of indels in the CDS region was 1872 while it is 3240 in the IGS, almost 2 times more than the amount of the indels in the CDS region. For the SNPs, there is not much difference between the number of SNPs in CDS and in IGS, which are 190 and 259, respectively. Similar to the number of SNPs of the SSC region, most loci of indels were detected on the SSC region of seven *Durio* species and specifically, on the *ycf*1 gene. In addition, the *ycf*1 genes in the SSC region were detected with 970 loci of indels.

### 3.3 Repeat Sequences Analysis

#### 3.3.1 SSR Identification, Analysis and Primer Design

**FIGURE 2:**
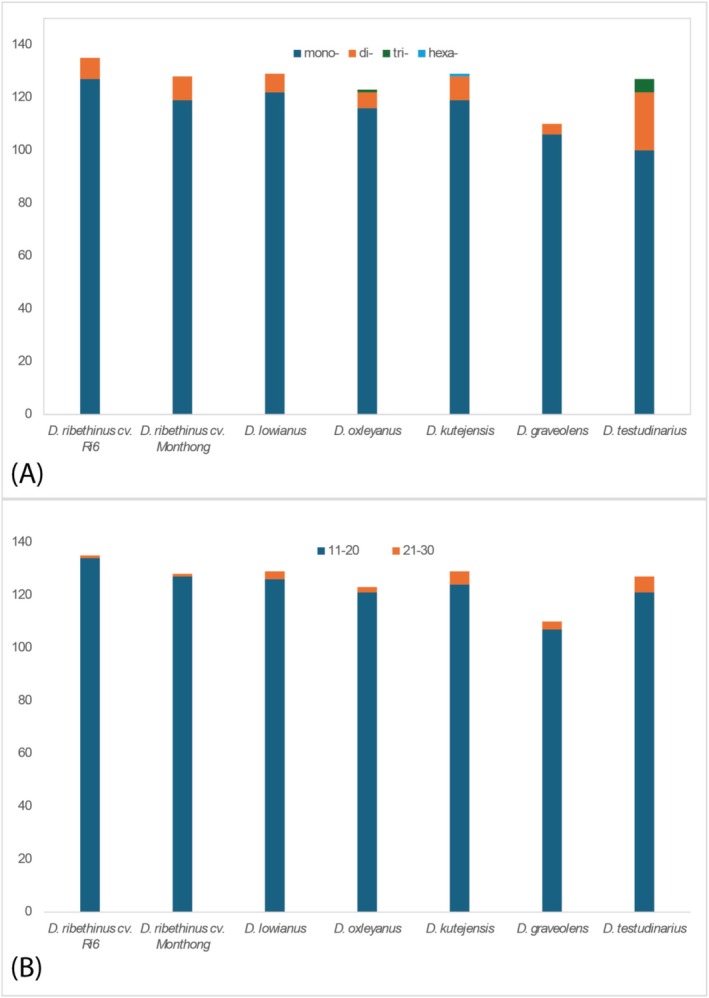
Number of SSR in chloroplast genomes of species in *Durio* genus. (A) Types of SSR. (B) Length of SSR.

The SSR sequences analysis results have shown differences between species in the *Durio* genus (Figure 2). The species with the most SSR sequences is *D. zibethinus* cv. Ri 6 with 135 sequences, and the least is *D. graveolens* with only 110 recorded. Although the tool was set up to find all six types of SSR, any tetra‐ or penta‐nucleotide has been recorded. The most abundant type of SSR is mono‐nucleotide, with 809 cases among seven species, and the second most abundant is di‐nucleotide, with 65 cases. For much rarer cases, only two species (*D. oxleyanus* and *D. testudinarius*) were detected with trinucleotide, and only *D. kutejensis* is detected with one hexanucleotide. For the size of the repeat, most of them vary from 11 to 20 bp, of which the species with the most SSR detected is *D. zibethinus* cv. Ri 6, followed by *D. zibethinus* cv. Monthong and *D. lowianus*, which are 134, 127, and 126, respectively. Besides that, SSR with the size from 21 to 30 bp was also detected in low amounts ranging from one to six.

The SSR data of *Durio zibethinus* cv. Ri6 was selected for primer design specific on SSRs using Primer3Plus and evaluated the rating score using NetPrimer. Among 135 SSR markers of *D. zibethinus* cv. Ri6 detected with lengths ranging from 10 to 330 bp, only some of the SSRs can be utilized for primer designing. Consequently, a total of 13 different primer pairs were able to be designed with high rating scores with the amplicon size varying from 236 to 962 bp (Table 2). The melting temperatures were provided bioinformatically. These primer pairs can potentially be used for phylogenetic relationship studies or identifying different *Durio zibethinus* varieties. For instance, as for applying, theoretically, the same primer pair *Durio zibethinus* cv. Ri6 for various cultivars, specific DNA profiles could be acquired. Furthermore, such primers should be applied in experimental validation to characterize the genetic variation. In the previous publication from Lin et al. (2022), the SSR markers that have been identified and developed will serve as a cornerstone for molecular breeding, supporting marker‐assisted selection, quantitative trait locus (QTL) mapping, and the discovery and validation of candidate genes.

**TABLE 2:** Thirteen primer pairs from SSR data of *D. zibethinus* cv. Ri6 sequences, melting temperature (*T*_m_) and amplicon length.

Markers	Direction	Sequence (5′ → 3′)	T_m_ (°C)	T_a_ (°C)	Amplicon length
CpDz01	Forward	TCCGCGATCTAGGCATAGGT	59.83	55.2	236
	Reverse	ACCTGCTATGCACAAAACACA	56.61		
CpDz02	Forward	TCTCTTTTGATGGAAAGGGAGCA	62.95	57	284
	Reverse	TTGGTAATTGGTCAAGCTCGA	58.41		
CpDz03	Forward	CGCAATTCTCTCCGGTGGTTA	62.55	59	334
	Reverse	TTTTGATGGATTCGGCGGAT	62.58		
CpDz04	Forward	TGAACAACCGTACAGGCATT	56.26	57	379
	Reverse	CCCATCTCAGGCGTCACAAA	61.48		
CpDz05	Forward	TGATGGAGAGTGAGTGCGAA	56.81	57	393
	Reverse	TACCCCTCTCCCTCCATCAA	59.09		
CpDz06	Forward	CGATCAATCCCTTTGCCCCT	63.52	62	423
	Reverse	TTCGGGATTTTCCTTGGGGG	65.55		
CpDz07	Forward	GGTTCGGTGGACAAAGCAAA	60.78	60	428
	Reverse	GCCAAAAGCCCCTTATCGGA	63.85		
CpDz08	Forward	AAAGCCAGTTGTTGCTGCTG	58.92	57	444
	Reverse	AAGGAGTATTGATTATCGAACCGA	59.46		
CpDz09	Forward	GTGGTGGAGGAACTGGATCG	59.74	58	481
	Reverse	TTCGGGGGTCCAAGAGTTTT	60.93		
CpDz10	Forward	GTCCAGTAGCGACAACCGTT	57.5	57	519
	Reverse	ACCAATCAGAATTGCCTCCCA	61.69		
CpDz11	Forward	TCGTTGCATTGAATGAAATCGACT	63.67	58	547
	Reverse	CAGCCGTTCCTATACCTGTCA	57.85		
CpDz12	Forward	AGAGAGATCCACCAGGGCAA	62.77	59	696
	Reverse	AGAGAGATCCACCAGGGCAA	59.19		
CpDz13	Forward	AAAATGACCCCTCCCACGAA	61.51	59	962
	Reverse	ACTTTCTTCAGTTCAGGGCGA	59.1		

**FIGURE 3:**
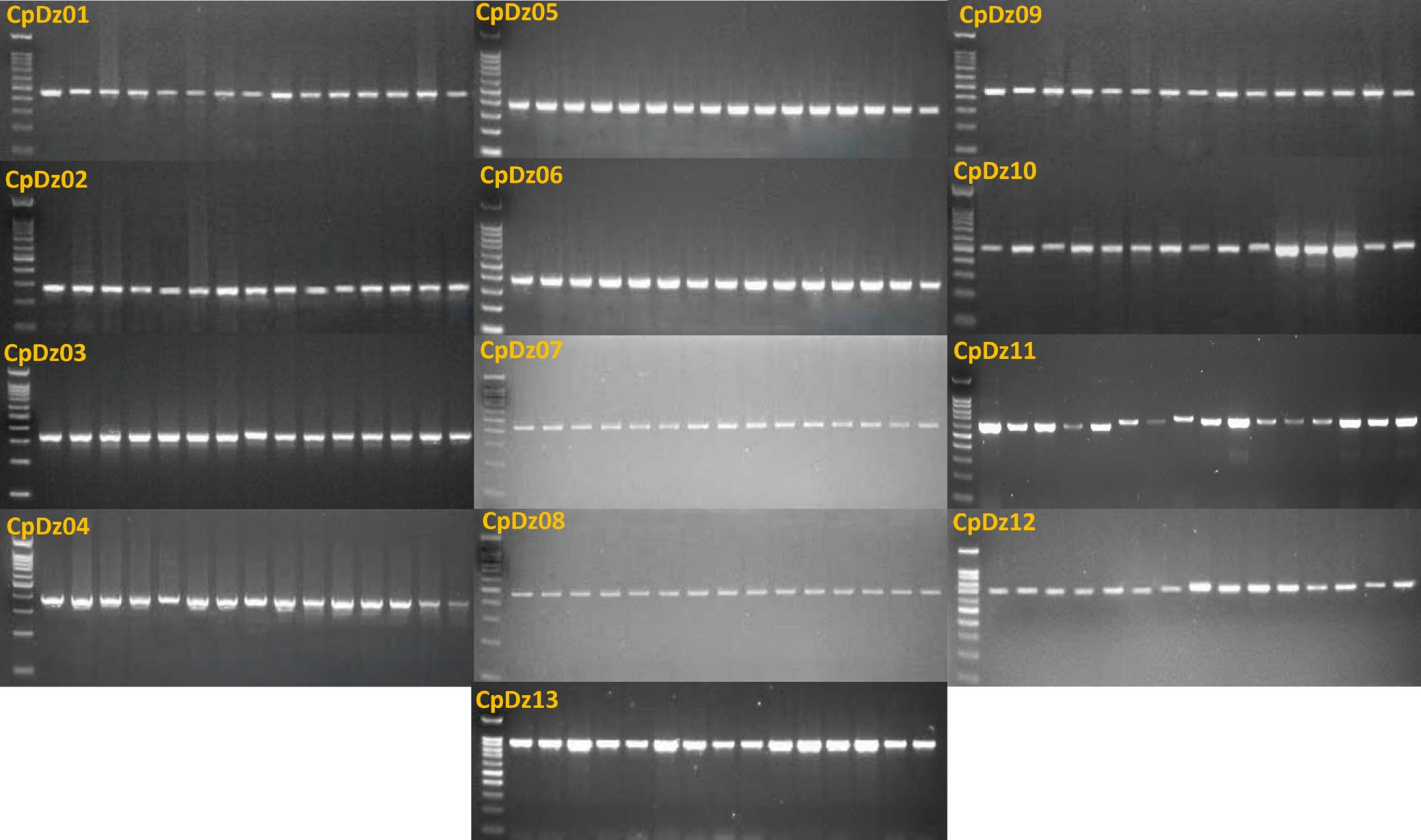
PCR amplification profiles of 13 CpDz chloroplast EST‐SSR primer pairs (CpDz01—CpDz13) across 15 durian trees (Lane 2–6: Ri6 cultivar, Lane 7–11: Musang King cultivar, Lane 12–16: Monthong cultivar).

The primer pairs were evaluated via PCR using 15 durian DNA samples representing three cultivars—Ri6, Monthong, and Musang King (five trees per cultivar). The amplicon sizes observed on the agarose gel matched the predicted fragment lengths (Figure 3); however, minor cultivar‐specific size variations were detected with primer sets CpDz10 and CpDz11.

#### 3.3.2 Tandem Repeat Identification and Analysis

**FIGURE 4:**
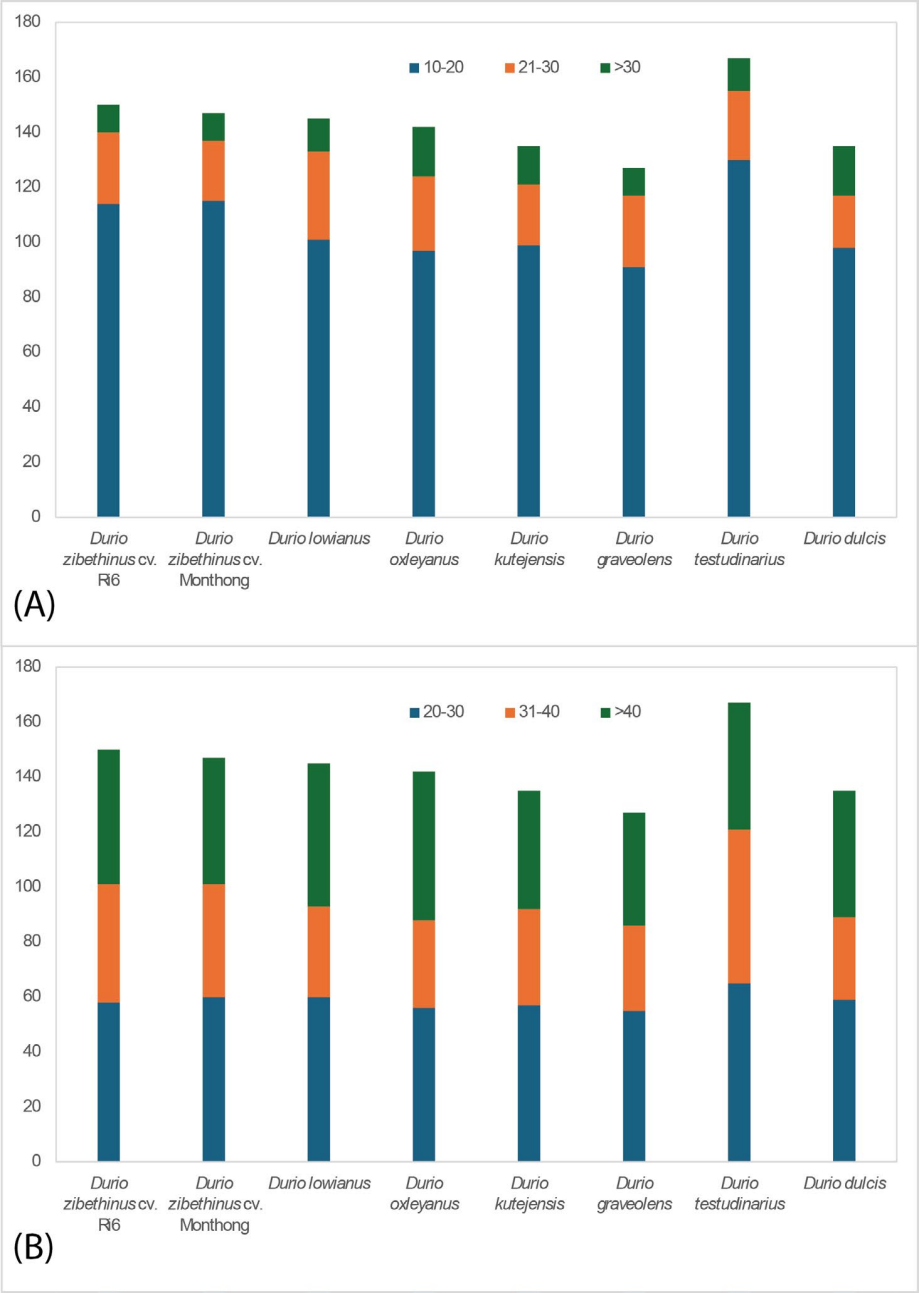
Number of long repeats in chloroplast genomes of species in the *Durio* genus. (A) Repeat unit size. (B) Length of repeat.

For the tandem repeat sequences analysis results between species in the *Durio* genus (Figure 4). For the size of the repeat unit, for the group having a 10–20 bp repeat unit, two cultivars of the same species *D. zibethinus* have an approximate amount which is 114 for the Ri6 cultivar and 115 for the Monthong cultivar. The species with the most amount of repeat units of 10–20 bp is *D. testudinarius*, and the species with the least amount of repeat units of 10–20 bp is *D. graveolens* . For the group of 21–30 bp repeat units, it is much less than the group of 10–20 bp repeat units, which is between 22 and 32. For the group having more than 30 repeat units bp, most of the species do not have much of this group as *D. zibethinus* cv. Monthong, *D. zibethinus* cv. Ri6, and *D. graveolens* only have 10 tandem repeats that have over 30 bp repeat units. The CDS of all *Durio* species that were detected with the tandem repeat are *acc*D, *ndh*A, *ndh*F, *ycf*1, and *ycf*2. Other CDS regions that were also detected in most *Durio* species with the tandem repeat were *ycf*3, *rps*3, and rps19. Only some *Durio* species detected the tandem repeat in certain CDS regions, including *ndh*B (in *D. oxleyanus*), *rpo*A (in *D. lowanius*), *rps*2 (in *D. graveolens* ), and *rps*18 (in *D. testudinarius*).

*Durio* chloroplast genomes vary markedly in their tandem repeats: the *D. zibethinus* plastome harbors 144 simple sequence–repeat loci, while assemblies from multiple varieties show the canonical ~25 kb inverted repeat highly reduced or even absent, leaving only short, copy number–variable remnants (Shearman et al. 2020). Such repeats sit beside promoters or fold into 3′ stem loops that define transcript ends; altering their length changes promoter spacing, RNA cleavage sites, and mRNA stability, so expansions or contractions can quantitatively tune plastid gene expression and, in turn, photosynthetic, pigment biosynthesis, or stress response traits among Durio species (Jacobs and Kück 2011).

### 3.4 Phylogenetic Tree Analysis

#### 3.4.1 *Durio* Genus Phylogenetic Tree

To analyze the phylogenetic relationship of current sequences of *Durio* species on the NCBI database. Two types of models were used to reconstruct the phylogenetic tree of the species in the *Durio* genus, which were the SNPs/indels model and CDS model. From the calculation of Maximum Likelihood evolutionary models of the IQ‐Tree web server, the model GTR + F + R2 was indicated as the best‐fit model according to the BIC for the CDS‐based model; for the SNPs/indels‐based model, the K3Pu + F + G4 model was chosen as the best‐fit model.

**FIGURE 5:**
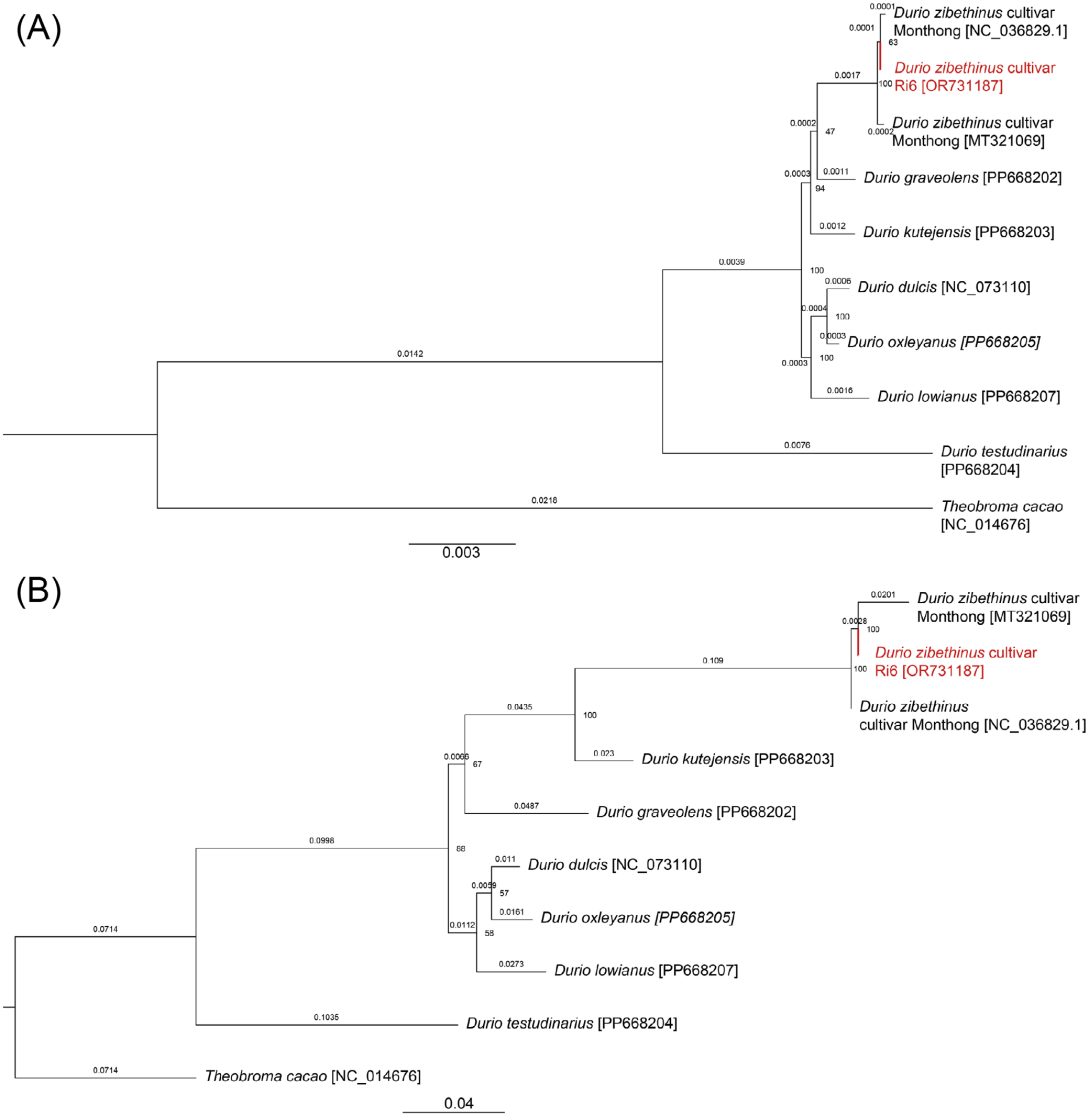
ML phylogenetic tree of nine complete chloroplast DNA sequences of species in *Durio* genus and *Theobroma cacao* as an outgroup. (A) CDS‐based model. (B) SNPs/indels‐based model.

From Figure 5A, which is the phylogenetic tree reconstructed by the CDS region, *Durio zibethinus* cv. Ri6 is closely related to other *Durio zibethinus* cultivars, particularly *Monthong* (NC_036829.1 and MT321069). The bootstrap value of 63 supports the close relationship among these cultivars. The branch lengths in this tree indicate minimal genetic divergence within the *Durio zibethinus* group. The genetic distance between *Durio zibethinus* cv. Ri6 and other *Durio zibethinus* cultivars is very small, reflecting their high level of genetic similarity in terms of the coding sequence regions. The tree also reflects a general trend of minimal divergence within the *Durio zibethinus* group, suggesting that coding sequences are highly conserved among these cultivars. The close clustering of *Durio zibethinus* cv. Ri6 with *Monthong* indicates that these cultivars likely share a very recent common ancestor. The *Durio zibethinus* cultivars, including Ri6, form a distinct cluster separate from other *Durio* species like *Durio graveolens*, *Durio kutejensis*, *Durio dulcis*, *Durio oxleyanus*, and *Durio lowianus*. The genetic distances between *Durio zibethinus* cultivars are minimal compared to the distances between different *Durio* species, as shown by the branch lengths.

Similarly, in Figure 5B, *Durio zibethinus* cv. Ri6 also clusters closely with the same *Durio zibethinus* cultivars *Monthong* (NC_036829.1 and MT321069). Unlike Figure 4A, the bootstrap value of 100 at the node where Ri6 and *Monthong* split strongly supports this relationship. The branch lengths in this tree are slightly longer than in the CDS‐based tree, suggesting a higher degree of genetic differentiation captured by SNPs and indels. The genetic distance between *Durio zibethinus* cv. Ri6 and other cultivars is slightly greater compared to the CDS‐based tree, indicating that SNPs and indels capture more genetic variability. This tree suggests that while the cultivars are still very closely related, there are detectable differences in their SNP and indel profiles, which may not be as apparent when analyzing only coding sequences. *Durio zibethinus* cultivars, including Ri6, form a distinct cluster that is separate from other *Durio* species, such as *Durio kutejensis*, *Durio graveolens*, *Durio dulcis*, *Durio oxleyanus*, and *Durio lowianus*. The branch lengths between *Durio zibethinus* cultivars are relatively short, suggesting a close evolutionary relationship, while the branch lengths separating them from other *Durio* species are longer, indicating greater genetic divergence.

*Theobroma cacao* is used as an outgroup, which is used to root the phylogenetic tree to analyze the similarity and grouping of species in the same genus. And similar to the previous tree, it is clearly separated from the *Durio* species, demonstrating the distinct evolutionary lineage of *Theobroma cacao* compared to the *Durio* genus. Although, the distance between *Theobroma cacao* , an outgroup, of two phylogenetic trees reconstructed by different methods showed different length.

Overall, both the CDS‐based and SNP/indel‐based phylogenetic trees demonstrate that *Durio zibethinus* cv. Ri6 is closely related to other *Durio zibethinus* cultivars, particularly *Monthong*. The consistency in their placement across both trees indicates strong genetic ties, likely due to a recent common ancestor or selective breeding. However, the SNP/indel‐based tree provides slightly more resolution in terms of genetic differentiation between the cultivars, suggesting that this method captures additional genetic variation that may not be evident in CDS analysis alone. This suggests that while coding sequences are highly conserved, there is additional genetic variation in the non‐coding regions or minor coding changes that are better captured through SNPs and indels. The higher bootstrap values in the SNP/indel‐based tree also suggest greater confidence in the relationships among the cultivars, likely due to the increased number of informative sites provided by SNPs and indels. Therefore, the SNP/indel model was applied to reconstruct and analyze further the phylogenetic relationship of the *Durio* genus within the Malvaceae family.

#### 3.4.2 Malvaceae Family Phylogenetic Tree

**FIGURE 6:**
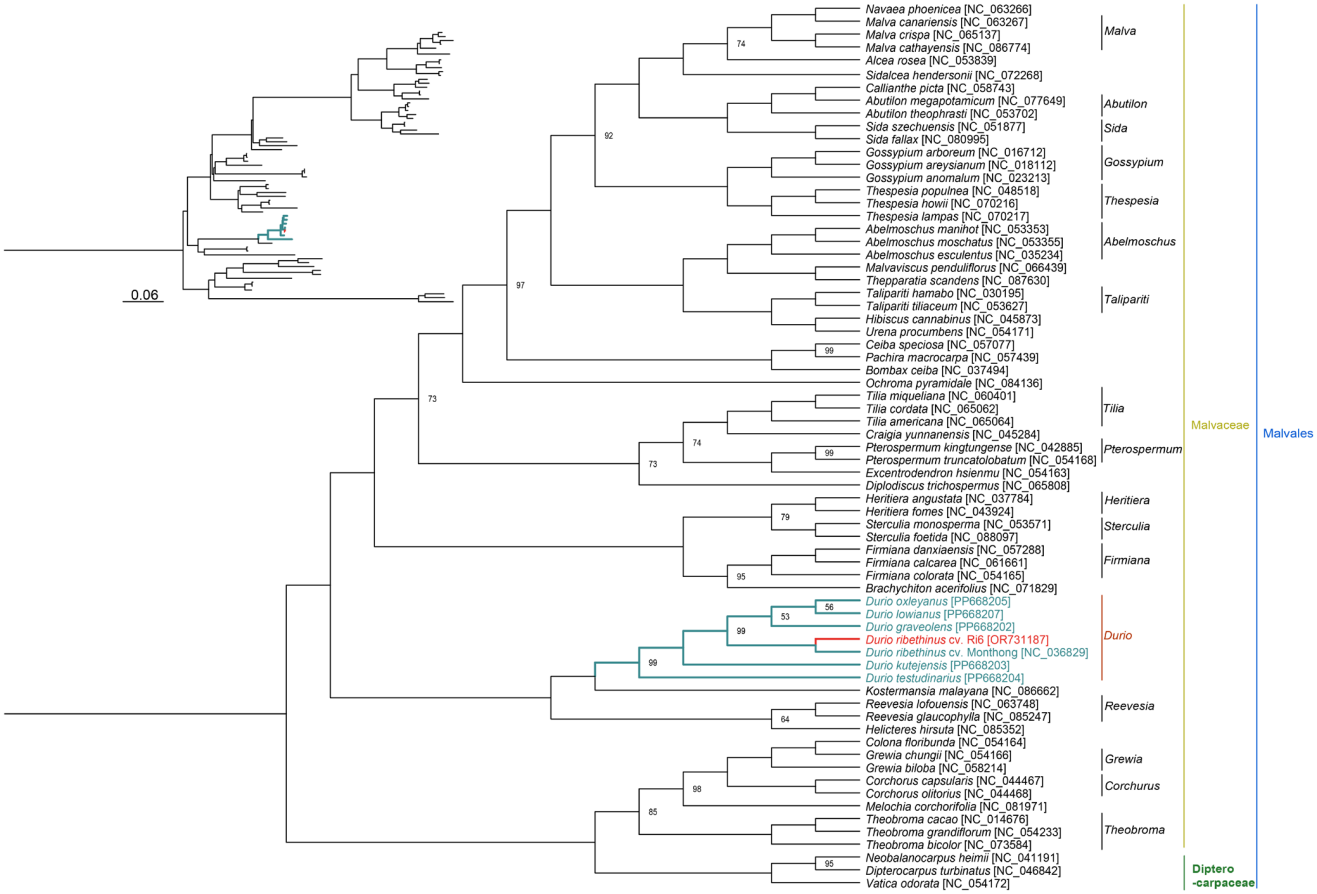
ML phylogenetic tree of 69 complete chloroplast DNA sequences of Malvaceae family based on SNPs/indels. The left tree is a phylogram which shows the true distance of each clade. The right tree is a cladogram for better observation.

From the analyzing result of the phylogenetic trees reconstructed by two different models, the CDS‐based model and the SNPs/indels‐based model, the SNPs/indels‐based model was selected to reconstruct the phylogenetic tree of the Malvaceae family (Figure 6). From the calculation of the IQ‐Tree web server, the best‐fit ML evolutionary model is TVM + F + R4 based on the BIC.

For the expansion analysis of the phylogenetic relationship of the *Durio* genus, the Malvaceae tree was reconstructed using SNPs and indels, providing a detailed picture of the genetic relationships within the family, including the *Durio* genus and other related genera. The tree's branching patterns and bootstrap values indicate the evolutionary relationships and the confidence level in those relationships. Three species in the Dipterocarpaceae family, *Neobalanocarpus heimii* (NC_041191), *Dipterocarpus turbinatus* (NC_046842), and *Vatica odorata* (NC_054172), were used as an outgroup to root the phylogenetic tree and successfully separate themselves from other genera and species in the Malvaceae family.

From the phylogram, we can clearly see that there are six separate clades, excluding the outgroup. The first clade includes at least 7 genera, including *Malva, Abutilon, Sida, Gossypium, Thespesia, Abelmoschus*, and *Talipariti*. The second clade includes the species *Ceiba specosa, Pachira macrocarpa*, and *Bombax ceiba*. The third clade includes at least two genera, *Tilia and Pterospermum*. The fourth clade includes at least three genera, *Heritiera, Sterculia*, and *Firmiana*. The fifth clade is the clade of species from the *Durio* and *Reevesia* genera. The sixth clade includes species from the genera of *Grewia, Coichuris*, and *Theobroma*.

The *Durio* genus forms a well‐defined monophyly clade, which is supported by a high bootstrap value of 99, indicating strong confidence in the grouping of these species within this genus, as well as the similarity in genetic differentiation. The *Durio* genus forms a distinct and well‐supported clade within the tree, situated closer to certain genera like *Reevesia* and *Firmiana* than to others like *Theobroma* and *Grewia*. The position and branch lengths provide clues about the relativeness. *Reevesia* is one of the genera most similar to *Durio* in terms of clade location and branch lengths. The close closeness of these clades reflects a very recent common ancestor, implying that *Durio* and *Reevesia* diverged more recently than other taxa. The shorter branch lengths between *Durio* and *Reevesia* show that fewer genetic changes have occurred between these groupings than others, supporting the notion of a tighter connection.

In summary, for the *Durio* genus, the chloroplast genome is a useful tool in distinguishing between domesticated species, or cultivars, with another domesticated one and between domesticated species and wild species. These differences are minor yet many, when combined, contributed to the evolution of the species, or the whole genus, and can clearly be observed through the reconstructed phylogenetic tree. The analysis of the reconstructed phylogenetic tree has shown that the differences analyzed by the SNPs/indels are well more observable and provide slightly more resolution in terms of genetic differentiation, which was visualized via the comparison between two models of the phylogenetic tree. Although the currently sequenced species in the *Durio* genus have shown us a clear picture and their location on the phylogenetic tree based on the chloroplast genomes, more samples of the *Durio* genus should be collected to complete the phylogenetic relationship between species in the genus, as well as cover the gap in the Malvaceae family.

## Author Contributions

**Tran Gia Huy:** data curation (equal), investigation (equal), methodology (equal), writing – original draft (equal), writing – review and editing (equal). **Tran Van Be Nam:** conceptualization (equal), data curation (equal), funding acquisition (equal), project administration (equal), resources (equal), writing – review and editing (equal). **Nguyen Pham Anh Thi:** conceptualization (equal), data curation (equal), funding acquisition (equal), project administration (equal), resources (equal), writing – review and editing (equal). **Pham Thanh Phuc:** conceptualization (equal), data curation (equal), writing – review and editing (equal). **Do Tan Khang:** data curation (equal), formal analysis (equal), funding acquisition (equal), writing – review and editing (equal).

## Conflicts of Interest

The authors declare no conflicts of interest.

## Data Availability

The chloroplast genome sequences supporting this study's results are freely available in NCBI's GenBank (https://www.ncbi.nlm.nih.gov) under the accession numbers provided in Table 1.
